# Integrated Transcriptomic and Metabolomic Analysis Reveals Key Genes in Fatty Acid and Flavonoid Biosynthesis in Macadamia Nut (*Macadamia integrifolia*)

**DOI:** 10.3390/biology15181589

**Published:** 2026-09-09

**Authors:** Qiujin Tan, Xiaokang Fan, Chunheng Zhou, Xiyun Huang, Zhenzhen Pan, Yuanrong Wei, Xiaozhou Yang, Wenlin Wang

**Affiliations:** 1 Guangxi Key Laboratory of Smart Agriculture for Characteristic Horticultural Crops, Chongzuo Field Integrated Scientific Observation and Research Station of the Ministry of Agriculture and Rural Affairs, Guangxi South Subtropical Agricultural Science Research Institute, Longzhou 532415, China; tqiujin110@gxaas.net (Q.T.); zhouchunheng@foxmail.com (C.Z.); huangxiyun04@gxaas.net (X.H.); panzhenzhen0302@163.com (Z.P.); m18154714950@163.com (Y.W.); yxz18848966680@163.com (X.Y.); 2 College of Tropical Agriculture and Forestry, Hainan University, Danzhou 571737, China; fanxiaokang2023@163.com

**Keywords:** macadamia nut, transcriptome, MibHLH46, fatty acid, flavonoid

## Abstract

bHLH transcription factors are central to plant development and to the metabolism of fatty acids and flavonoids. Through the transcriptomic, metabolomic, and phytohormone/elemental profiling of two macadamia varieties with divergent fatty acid content, ‘Guire No. 1’ (GR1) and ‘A4’, we identified 142 MibHLH transcription factors, classified into 21 subfamilies, of which 14 show collinearity with Arabidopsis AtbHLH members. MibHLH46 was markedly differentially expressed, and its expression strongly correlated with that of fatty acid biosynthesis genes. Promoter analysis further revealed abundant MYC and G-box elements in fatty acid and flavonoid biosynthetic genes. Comparative metabolomics showed that ‘GR1’ accumulates higher levels of several mineral elements (Fe, Ca, Mg) and abscisic acid (ABA) than ‘A4’. These transcriptomic trends were confirmed by qRT-PCR, which validated the up-regulation of genes related to fatty acid oxidation, α-linolenic acid metabolism, and the flavonoid pathway in ‘GR1’. We propose that MibHLH46, as a candidate regulator, contributes to kernel fatty acid and flavonoid metabolism by transcriptionally regulating key biosynthetic genes, providing a basis for identifying regulatory genes in macadamia lipid metabolism.

## Simple Summary

Macadamia nuts are valued for their healthy fats, but the genetic control of fat and flavonoid content is not fully understood. This study compared two macadamia varieties, ‘GR1’ (higher fat and flavonoid levels) and ‘A4’ (lower levels), using gene activity and metabolite profiling. We identified 142 bHLH transcription factor proteins that regulate gene expression and found that one, MibHLH46, was much more active in ‘A4’. Its activity correlated with genes involved in fat and flavonoid production. ‘GR1’ also had higher levels of minerals like iron, calcium, and magnesium, and the hormone abscisic acid, which may influence these pathways. Our findings suggest that MibHLH46 is a candidate negative regulator of fat and flavonoid synthesis in macadamia kernels. This discovery provides a target for breeding programs in developing macadamia varieties with improved nutritional quality, such as higher healthy fat content, benefiting human health and the agricultural economy.

## 1. Introduction

*Macadamia* nut, also known as *Macadamia integrifolia* Maiden and Betche, is an evergreen fruit tree of the family Proteaceae, native to Australia [1]. *Macadamia* nut kernels are rich in oil/fat [2] and can be used as medicine and food [3]. *Macadamia* nut kernels are renowned for their high fat content, which is predominantly composed of beneficial unsaturated fatty acids. Clinical studies indicate that daily consumption of 40–90 g *Macadamia* nuts providing 15% of energy for 4 weeks significantly lowered plasma total cholesterol and bad cholesterol LDL-C while increasing good cholesterol HDL-C in hypercholesterolemic men [4]. The high oleic acid content also contributes to better oxidative stability. Compared to oils rich in polyunsaturated fatty acids (PUFAs), *Macadamia* nut oil produces fewer toxic peroxidation products during thermal oxidation, and its extracts, especially its oleic acid-rich extracts, exhibit anti-inflammatory effects by inhibiting the expression of inflammatory mediators in immune cells [5,6]. Analysis shows that *Macadamia* nuts contain approximately 78.52% fat, with unsaturated fatty acids accounting for 69.03% of the total fatty acids. The monounsaturated fatty acid oleic acid (C18:1) is the most abundant, reaching up to 49.24%. The fatty acid profile is significantly influenced by both varieties and developmental stage. Studies on three major varieties, namely ‘GR1’, ‘O.C.’, and ‘HASE695’, in Guangxi, China, across 155–195 days after full bloom revealed dynamic changes in fatty acid composition, helping to determine the optimal harvest period for quality and nutritional value. The unique fatty acid profile, rich in monounsaturated fatty acids (MUFAs) and plant sterols, contributes to cardiovascular health.

Research on *Macadamia* and other plants has identified numerous genes central to these metabolic pathways. Genomic studies of *Macadamia integrifolia* and *M. tetraphylla* have revealed expansions in key gene families related to fatty acid biosynthesis and genome duplication events, providing a genetic basis for its high oil content [7]. Specific genes identified include those for acyl-ACP desaturases, which influence fatty acid unsaturation patterns [8]; 3-ketoacyl-CoA synthases (KCS), which are involved in very-long-chain fatty acid (VLCFA) elongation [9,10]; and genes associated with cyanogenic glycoside metabolism, which shares precursors with some amino acids [11,12]. Transcriptome analyses comparing *Macadamia* varieties with different shell thicknesses identified thousands of new genes and highlighted the crucial role of the MiMYB transcription factor family, particularly subfamilies S4, S6, and S7, in flavonoid biosynthesis and shell formation [13]. Furthermore, integrated metabolomic and transcriptomic profiling of *Macadamia* shells has mapped phenolic and flavonoid biosynthetic pathways and their regulatory mechanisms across different developmental stages [14].

The basic helix–loop–helix (bHLH) transcription factor family is a major regulator of both flavonoid and fatty acid metabolism. bHLH proteins are core components of the MBW complex (MYB-bHLH-WD40), which coordinately activates the genes of the flavonoid/anthocyanin pathway. This has been extensively reviewed and demonstrated in plants like *Arabidopsis*, petunia, tea tree, and *Macadamia* [15,16]. For instance, in *Arabidopsis*, a bHLH factor TT8 controls flavonoid accumulation in seeds through a feedback loop involving TTG1 and MYB partners [17]. In *Macadamia*, bHLH factors are implicated alongside MYBs in regulating shell-related flavonoid pathways [13]. bHLH transcription factors also directly regulate lipid metabolism. In *Arabidopsis*, *bHLH129* is an ABA-downregulated transcriptional repressor that influences root growth and ABA sensitivity, potentially connecting hormone signaling with growth–metabolism balance [18]. More directly, CFLAP1 and CFLAP2 are two bHLH factors that cooperate with AtCFL1 to regulate the expression of genes in the fatty acid, cutin, and wax biosynthetic pathways, affecting cuticle development [19]. In the microalga *Nannochloropsis salina*, overexpression of the *NsbHLH2* transcription factor increased biomass and fatty acid methyl ester (FAME) productivity, especially under nitrogen limitation [20]. bHLH factors often act as hubs integrating environmental cues with metabolic reprogramming. They are key players in abiotic stress responses [21,22], shade avoidance [23], and hormone signaling, such as jasmonic acid (JA) signaling mediated by MYC2–JAZ interactions [24]. This permits them to coordinately modulate both defensive flavonoid synthesis and adaptive changes in membrane lipids under stress. 

It is evident that it is necessary to screen the key regulatory genes that control the synthesis of fatty acids in *Macadamia* nuts through the combined analysis of transcriptome and metabolome, providing genetic resources for the breeding of high-yield and high-quality varieties.

## 2. Materials and Methods

### 2.1. Materials

Two *Macadamia* nut varieties, abbreviated as ‘GR1’ and ‘A4’, with significant differences in kernel fatty acid contents, were used in this study. The fruit samples of the two varieties were obtained from the *Macadamia* germplasm resource nursery (106°79′22″ E, 22°34′13″ N) of the Institute of Subtropical Agricultural Sciences in Guangxi, China. The fruit samples were collected on the same day, ensuring equal maturity. The samples were collected in liquid nitrogen and stored in a −80 °C ultra-low-temperature freezer (Thermo Scientific, Waltham, MA, USA). Then, their ash content, crude fat content, crude protein content, and total sugar contents were measured based on the dry weight (DW) according to the Chinese standards GB/T 6438—2025 [25], GB/T 6433—2025 [26], GB/T 6432—2018 [27], and GB/T36056—2018 [28] (https://www.ndls.org.cn/ (accessed on 20 May 2025), respectively. Three biological replicates were performed for each sample. All the chemical reagents used for forage quality measurements were bought from Sangon Biotech Co., Ltd. (Shanghai, China) [29].

### 2.2. RNA Extraction and RNA-Seq Analysis

Total RNA was extracted from ‘GR1’ and ‘A4’ kernels with three biological replicates each using the RNAprep Pure Plant Plus Kit (Tiangen Biotech, Beijing, China), as described previously [13]. For RNA-seq analysis, kernel samples from each cultivar were collected with three independent biological replicates (*n* = 3). RNA concentration and integrity were assessed with a NanoDrop spectrophotometer (Thermo Scientific, Waltham, MA, USA), Qubit 2.0 Fluorometer (Invitrogen, Carlsbad, CA, USA), and Agilent 2100 Bioanalyzer (Agilent, Palo Alto, CA, USA). Only RNA samples of good quality were used for subsequent library construction. For library preparation, cDNA concentration and insert size were examined with Qubit 2.0 and Agilent 2100, and the precise library concentration was determined by qPCR; only libraries with a concentration greater than 2 nM were accepted. Qualified libraries were pooled according to the predetermined target data volume and sequenced on an Illumina sequencing platform. Raw reads were filtered to remove adapter sequences and low-quality reads, yielding clean data. The clean reads were mapped to the predefined reference genome, and the resulting mapped data were subjected to quality control assessments of insert size and sequencing randomness. RNA sequencing was performed using three independent biological replicates for each cultivar. After removal of adapter sequences and low-quality reads, a total of 39.34 Gb of clean data was obtained from the six libraries, corresponding to 5.92–8.57 Gb per sample. The percentage of bases with a Phred quality score ≥ Q30 ranged from 94.06% to 94.76%.

The clean reads were aligned to the *Macadamia integrifolia* reference genome (*Macadamia_integrifolia*. Submitted GenBank assembly: GCA_013358625.1, https://www.ncbi.nlm.nih.gov/datasets/genome/GCA_013358625.1/ (accessed on 1 May 2025)) using HISAT2. The overall mapping rates ranged from 90.35% to 91.37%, and uniquely mapped reads accounted for 85.63–86.98% of the total reads. Detailed sequencing and mapping statistics for each biological replicate are provided in Table S11. 

Basic analyses of the mapped data included gene expression quantification, alternative splicing analysis, novel gene prediction, and gene structure optimization. Differentially expressed genes (DEGs) were identified using a threshold of fold change ≥ 1.5 and FDR < 0.05 via the R package and subsequently subjected to GO and KEGG enrichment analyses. GO enrichment (biological process, molecular function, and cellular component) was performed with clusterProfiler using the hypergeometric test, and the results were visualized as histograms to determine the functions of the DEGs. The filtered sequences were aligned to the reference genome, and transcriptome assembly was carried out with StringTie v2.2.0 [30]. Transcription factors were identified based on gene annotations in the database and the PlantTFDB v5.0 platform (https://planttfdb.gao-lab.org/tf.php?sp=Mtr&did=Medtr1g086510.1 (accessed on 6 May 2025)) [31]. Multiple sequence alignments were generated with MUSCLE v5 [32], and a maximum-likelihood phylogenetic tree was constructed using IQ-TREE v2.3.4 [33] and visualized with FigTree v1.4.4.

### 2.3. Metabolomic Analysis of Macadamia Nut Varieties ‘GR1’ and ‘A4’ Kernel

Three independent biological replicates were also used for metabolomic profiling. *Macadamia* nut varieties ‘GR1’ and ‘A4’ kernel were used via metabolite extraction and identification and quantification of metabolites, following the method previously described [34]. Metabolite annotation was performed by matching detected features against the online METLIN database (https://ngdc.cncb.ac.cn/databasecommons/database/id/5907) (accessed on 1 June 2025), public databases, and Biomark’s in-house database, together with theoretical fragment information. The mass tolerances were set to within 100 ppm for precursor ions and 50 ppm for fragment ions. For differential metabolite screening, OPLS-DA was performed using the ropls package in R 3.6.1. Model reliability was evaluated via permutation testing (200 permutations), and VIP values were obtained through cross-validation. Differential metabolites were screened according to VIP ≥ 1 and *p* < 0.05 together with fold-change information. KEGG pathway enrichment analysis was performed using clusterProfiler, enrichplot, and ggplot2 in R 3.6.1, and *p* values from pathway enrichment analysis were adjusted using the false discovery rate (FDR) method. 

### 2.4. Determination of Mineral Elements and Phytohormones

To compare the mineral-nutrition and hormonal status of the two varieties, mineral elements and endogenous phytohormones in GR1 and A4 kernels were quantified. Mineral elements—Fe, Ca, Mg, K, P, etc.—were determined by inductively coupled plasma (ICP)-based spectrometry (Thermo Scientific, Waltham, MA, USA), and phytohormones (ABA, JA, IAA, etc.) were quantified by liquid chromatography–tandem mass spectrometry (LC-MS/MS) (Thermo Scientific, Waltham, MA, USA), both performed at a commercial analytical facility.

### 2.5. Quantitative Real-Time PCR (qRT-PCR) Validation

For qRT-PCR validation, RNA samples from three independent biological replicates were analyzed, with technical replicates performed for each biological replicate where applicable. To verify the transcriptomic expression patterns, qRT-PCR was performed on candidate genes involved in fatty acid oxidation, lipid and α-linolenic acid metabolism, and flavonoid biosynthetic using the same RNA samples. First-strand cDNA was synthesized from 1 μg total RNA using the RevertAid RT Kit (Thermo Scientific, Waltham, MA, USA) according to the manufacturer’s instructions. qPCR was carried out with ChamQ SYBR Color qPCR Master Mix (Vazyme Biotech Co., Ltd., Nanjing, China) on a CFX96 Touch Real-Time PCR Detection System (Bio-Rad, Hercules, CA, USA), using gene-specific primers with the macadamia malate dehydrogenase gene MiMADH as the reference. Each 20 μL reaction comprised 10 μL ChamQ SYBR Color qPCR Master Mix, 0.4 μL forward primer, 0.4 μL reverse primer, 1 μL cDNA template, and 8.2 μL ddH_2_O. The thermal program consisted of initial denaturation at 95 °C for 10 s; 40 cycles of 95 °C for 10 s and 60 °C for 30 s; and a melting-curve step at 95 °C for 15 s, 60 °C for 1 min, and 95 °C for 15 s. Relative expression was calculated via the 2^−ΔΔCT^ method [35], with ‘A4’ being the calibrator (relative expression = 1). Primer sequences are listed in Table S1. 

### 2.6. Statistical Analysis

The means and standard errors with all three biological replicates of the above experiments were analyzed via one-way variance analysis, and S-N-K’s test was used to test the homogeneity of variance. The graph was drawn by OriginPro 2021 (OriginLab Corporation, Northampton, MA, USA).

## 3. Results

### 3.1. Phenotypic and Metabolic Characterization of Macadamia Nut Varieties

**Figure 1:**
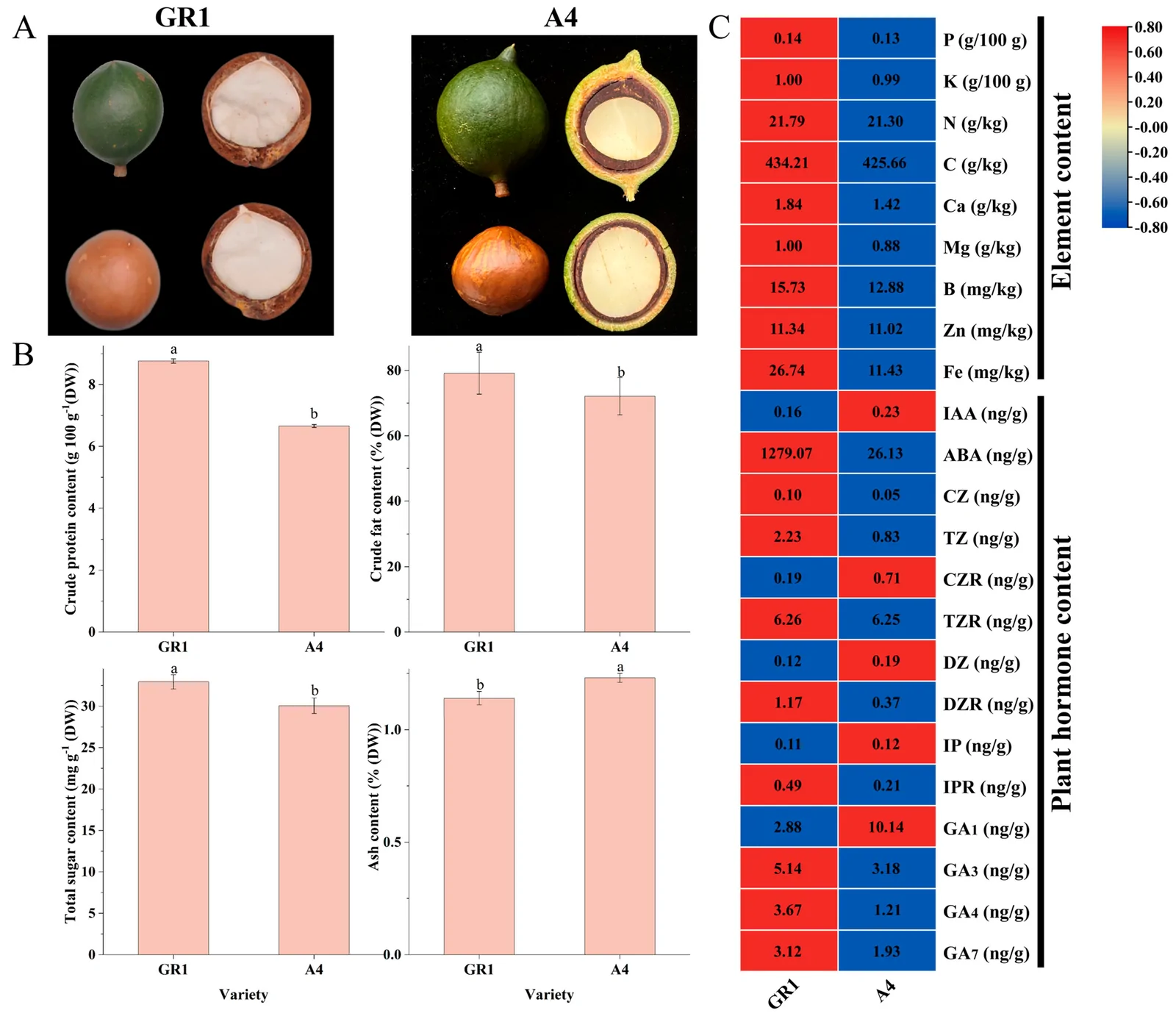
Phenotypic and metabolic characterization of *Macadamia integrifolia* varieties ‘GR1’ and ‘A4’. (**A**) Comparison of kernels between ‘GR1’ and ‘A4’. (**B**) Comparison of sugar and other metabolite contents between the two varieties. (**C**) Comparison of elemental and phytohormone contents between ‘GR1’ and ‘A4’. Data are presented as mean ± SD (*n* = 3). Different lowercase letters indicate statistically significant differences (*p* < 0.05).

The main nutrient contents in kernels of two main *Macadamia* nut varieties ‘GR1’ and ‘A4’ in Guangxi Province were analyzed. As shown in Figure 1, the crude protein content, crude fat content, and total sugar content in ‘GR1’ were 8.76 g 100 g^−1^ (DW), 79.16% (DW), and 32.94 mg g^−1^ (DW), which are significantly higher than the 6.66 g 100 g^−1^ (DW), 72.14% (DW), and 30.07 mg g^−1^ (DW) in ‘A4’. In contrast, the ash content was 1.14% in ‘GR1’, which was much lower than the 1.23% in ‘A4’ (*p* < 0.05). This indicates that the nutritional components in the kernels of the ‘GR1’ variety are better than those of the ‘A4’ variety (Figure 1B). The contents of nine mineral elements and fourteen endogenous hormones were comparatively quantified in the kernels of ‘GR1’ and ‘A4’. For mineral elements, the two varieties shared a largely similar overall profile, yet ‘GR1’ showed significantly higher levels for several elements. Iron (Fe) exhibited the most pronounced disparity. ‘GR1’ reached 26.74 mg kg^−1^, approximately 2.34 times that of ‘A4’ (*p* < 0.001). Calcium (Ca), magnesium (Mg), and phosphorus (P) were also elevated in ‘GR1’, and were found to be 1.30-fold, 1.14-fold, and 1.09-fold higher than those of ‘A4’ (*p* < 0.01), respectively, and carbon (C) and boron (B) were significantly higher in GR1 as well (*p* < 0.05). By contrast, potassium (K), nitrogen (N), and zinc (Zn) did not differ significantly between the two varieties. The most striking difference was in abscisic acid (ABA). ‘GR1’ accumulated ABA at 1279.07 ng g^−1^, roughly 48.95 times the level in ‘A4’ (*p* < 0.001), indicating a markedly stronger ABA signal in ‘GR1’ kernels. Cytokinin showed a divided pattern; trans-zeatin (tZ), dihydrozeatin riboside (DZR), isopentenyladenine riboside (IPR), and cis-zeatin (cZ) were significantly elevated in ‘GR1’ (1.8- to 3.2-fold those of ‘A4’ (*p* < 0.05)), whereas cis-zeatin riboside (cZR) was higher in ‘A4’ (about 3.75-fold that of ‘GR1’ (*p* < 0.05)). The remaining cytokinins (tZR, DZ, IP) showed no significant difference. Gibberellins (GAs) were predominantly higher in ‘GR1’, with GA_3_, GA_4_, and GA_7_ being 1.61-, 3.04-, and 1.62-fold higher than the corresponding values for ‘A4’ (*p* < 0.05), but GA_1_ was significantly higher in ‘A4’ (about 3.52-fold that of ‘GR1’ (*p* < 0.05)). Indole-3-acetic acid (IAA) did not differ significantly between varieties (*p* > 0.05) (Figure 1C).

Collectively, ‘GR1’ kernels are characterized by elevated Fe, Ca, and Mg, together with an exceptionally high ABA accumulation, revealing a systematic divergence in mineral nutrition and hormonal homeostasis from ‘A4’. These metabolic differences are plausibly associated with the divergent kernel quality, e.g., the lipid biosynthesis of the two varieties.

### 3.2. Transcriptome Screening in Macadamia Nuts ‘GR1’ and ‘A4’

**Figure 2:**
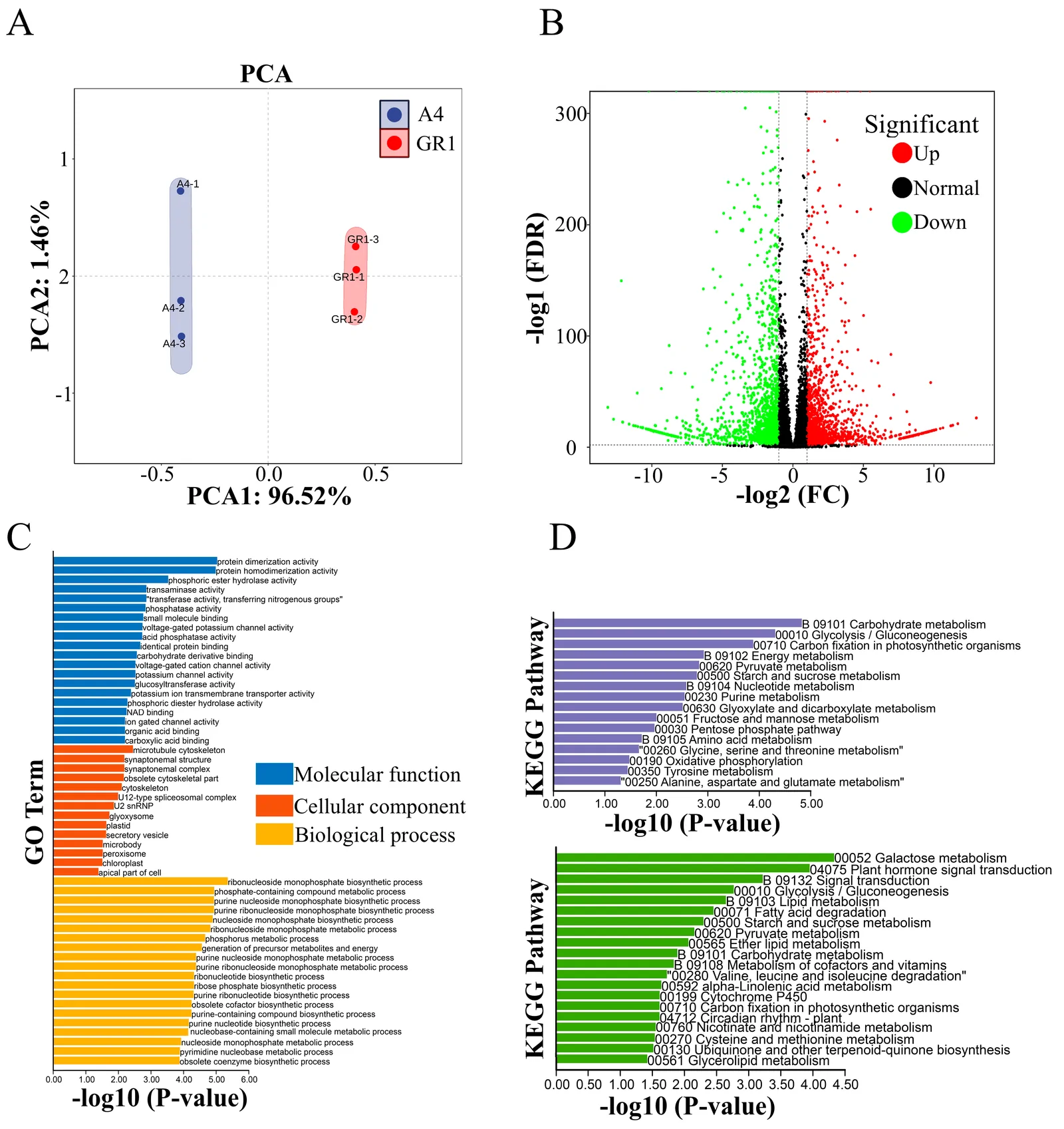
Transcriptome screening for DEGs in *Macadamia* nut varieties ‘GR1’ and ‘A4’: (**A**) PCA; (**B**) volcano plot of DEGs; (**C**) GO term analysis of DEGs; (**D**) KEGG pathway analysis of DEGs.

To characterize the transcriptional differences associated with the contrasting fatty acid profiles of the two macadamia cultivars, ‘GR1’ and ‘A4’, comparative transcriptomic analysis was performed on their kernels. Principal component analysis (PCA) (Figure 2A) shows the overall transcriptional variation between the samples of the two varieties. The first principal component (PCA1) explains 96.52% of the total variation, and the second principal component (PCA2) explains 1.46%. The samples of variety ‘A4’ and variety ‘GR1’ are clearly separated along the PCA1 axis. This indicates that there are significant differences in the overall transcriptional profiles between the two varieties, which provides a molecular basis for their differences in fatty acid content. A total of 18,484 genes were analyzed and the overall DEG rate is 18.75%. A total of 3465 genes shows significant differential expression—1667 up- and 1798 down-regulated—between the two varieties. Gene Ontology (GO) enrichment analysis is divided into three categories: molecular function (blue), cellular component (red), and biological process (yellow). For the molecular function category, genes are enriched in functions such as “protein dimerization activity”, “phosphoric ester hydrolase activity”, “transferase activity”, transferring nitrogenous groups”, etc. These functions may be related to the regulation of metabolic pathways and lipid metabolism at the molecular level. In the cellular component category, genes are enriched in components such as “cytoskeleton”, “synaptic vesicle”, “nucleus”, etc. These components may provide the structural basis for the differential expression of genes and the implementation of biological processes. In the biological process category, genes are enriched in processes such as the “ribonucleoside monophosphate biosynthetic process”, “phosphate-containing compound metabolic process”, “purine nucleotide metabolic process”, etc. These processes are likely to be related to the synthesis and metabolism of substances, including fatty acids, in the two varieties. KEGG pathway enrichment analysis is shown in two parts. For the upper part with purple background, genes are enriched in pathways such as “carbohydrate metabolism”, “glycolysis/gluconeogenesis”, “carbon fixation in photosynthetic organisms”, “pyruvate metabolism”, “energy metabolism”, “amino acid metabolism”, etc. These pathways are related to the energy supply and material synthesis for lipid metabolism. For the lower part with green background, genes are enriched in pathways such as “galactose metabolism”, “plant hormone signal transduction”, “lipid metabolism”, “fatty acid degradation”, “starch and sucrose metabolism”, “metabolism of cofactors and vitamins”, etc. The enrichment of lipid-related pathways, such as “fatty acid degradation”, directly indicates that the difference in fatty acid content between the two varieties is related to the differential regulation of lipid-metabolism pathways at the transcriptional level (Table S2).

**Figure 3:**
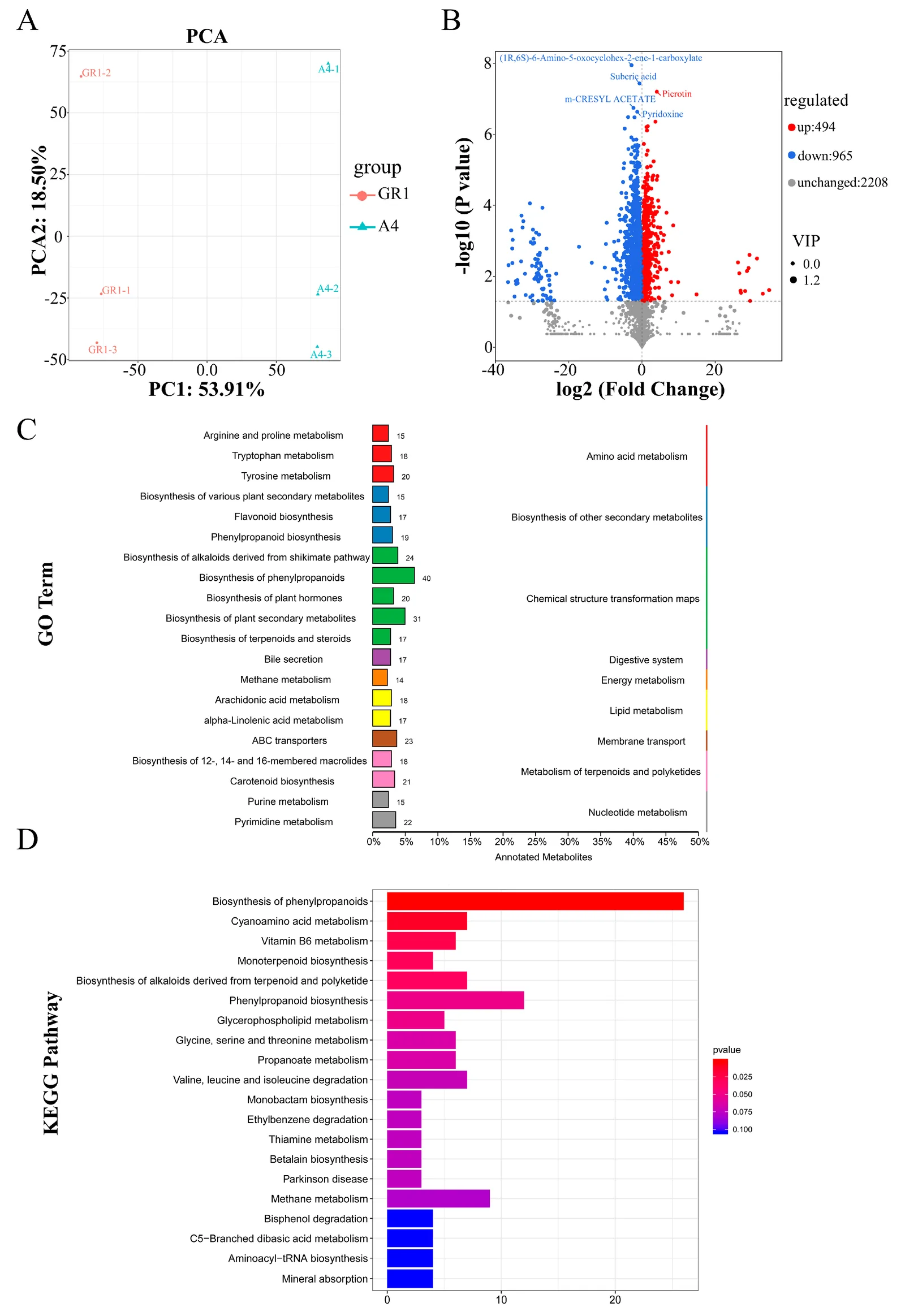
Metabolomic analysis identifies differential metabolites in *Macadamia* nut varieties ‘GR1’ and ‘A4’: (**A**) PCA; (**B**) volcano plot of metabolites; (**C**) GO term analysis of metabolites; (**D**) KEGG pathway analysis of metabolites.

As shown in Figure 3, the differential metabolites of the ‘GR1’ and ‘A4’ varieties are distributed in two quadrants (Figure 3A). The total number of identified metabolites were 3667, including 2208 unchanged, 494 up-regulated, and 965 down-regulated, respectively (Figure 3B). GO analysis revealed the extent of the biosynthesis of phenylpropanoids, the biosynthesis of plant secondary metabolites, and the biosynthesis of alkaloids derived from the shikimate pathway (40, 31, and 24, respectively) (Figure 3C). KEGG analysis revealed that phenylpropanoid biosynthesis and methane metabolism are ranked in the top 3 (Figure 3D). The PCA plot shows the distribution of samples from the two varieties. The first principal component (PC1) explains 53.91% of the total variance, and the second principal component (PC2) explains 18.50%. The samples of ‘GR1’ (red dots) and ‘A4’ (cyan triangles) are clearly separated in the PCA space. This separation indicates that there are significant differences in the overall metabolic profiles between the two *Macadamia* varieties (Figure 3A). The volcano plot is used to visualize the differentially regulated metabolites between the two varieties (Figure 3B). There are 494 metabolites with red dots above the significance threshold. There are 965 down-regulated metabolites with blue dots. The 2208 metabolites with gray dots do not change significantly between the two varieties. The distribution of these metabolites shows that there are a large number of metabolites with significant changes in abundance between ‘GR1’ and ‘A4’, suggesting extensive metabolic differences. The GO term enrichment analysis classifies the annotated metabolites into different functional categories. For amino acid metabolism, such as arginine and proline metabolism, tryptophan metabolism, and tyrosine metabolism, a certain proportion of metabolites are involved. For example, arginine and proline metabolism has 15 annotated metabolites, tryptophan metabolism has 18, and tyrosine metabolism has 20, respectively. For secondary metabolite biosynthesis, such as the biosynthesis of various plant secondary metabolites, flavonoid biosynthesis, and phenylpropanoid biosynthesis, a relatively large number of metabolites are involved. For instance, the biosynthesis of phenylpropanoids has 40 annotated metabolites, and the biosynthesis of plant secondary metabolites has 31 (Figure 3C). The KEGG pathway enrichment analysis identifies the metabolic pathways with significant differences between the two varieties. Pathways related to the biosynthesis of phenylpropanoids have a very low *p*-value, suggesting that this pathway is significantly different between ‘GR1’ and ‘A4’. Other significant pathways include cyanoamino acid metabolism, vitamin B6 metabolism, monoterpenoid biosynthesis, and the biosynthesis of alkaloids derived from terpenoid and polyketide (Figure 3D, Table S3). 

### 3.3. Identification of MibHLH Transcription Factor Family Members in Macadamia Nut

**Figure 4:**
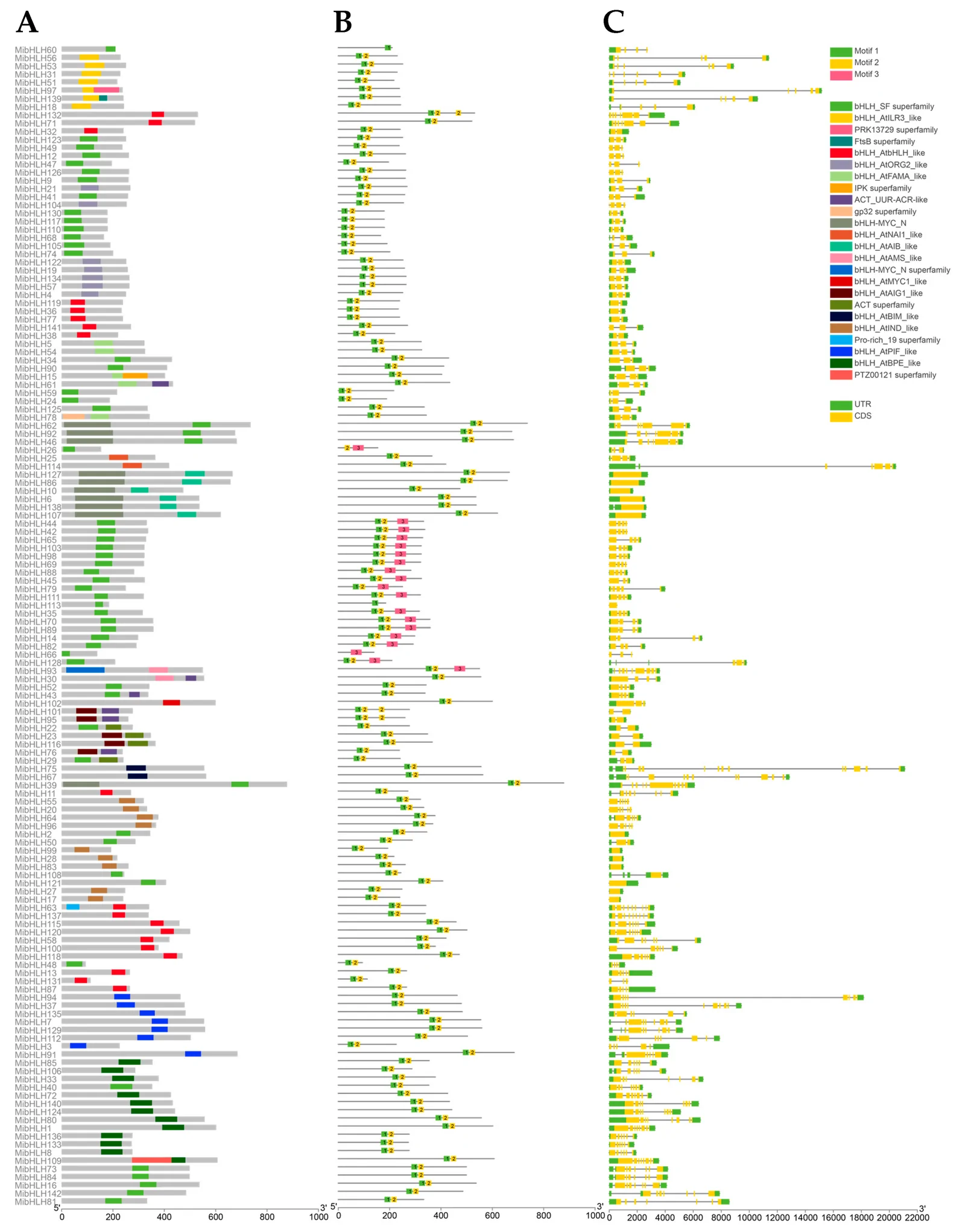
Comprehensive structural analysis of MibHLH transcription factors: (**A**) analysis of conserved protein domains within MibHLH proteins; (**B**) MEME motif analysis; (**C**) analysis of coding and non-coding regions.

The first panel showed an evolutionary relationship tree of the 142 transcription factors. The presence of specific, conserved motifs confirms the identity of these genes as transcription factors and facilitates their classification into subgroups based on their domain architecture (Figure 4A). The second panel identifies and maps conserved protein motifs within the transcription factors. Different colored boxes represent different conserved motifs, e.g., Motif 1, Motif 2, etc. (Figure 4B). The third panel displays the exon–intron organization of the 142 transcription factor genes. Each horizontal line represents a single gene. Exons are shown as thick colored bars. The variation in exon number and length among the genes is clearly visible. Introns are represented by the thin lines connecting the exons. The intron lengths also vary significantly between genes (Figure 4C, Table S4).

**Figure 5:**
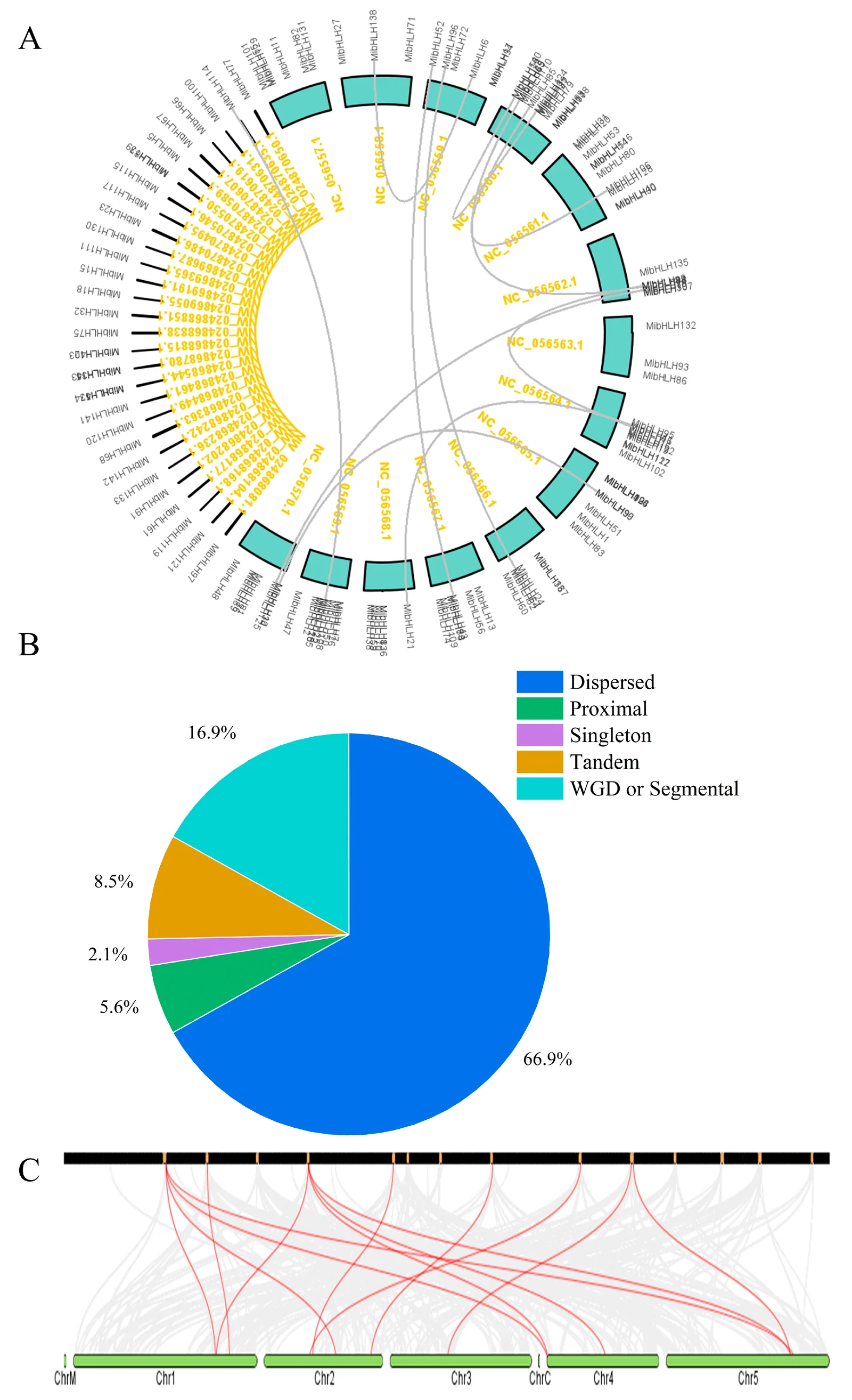
Phylogenetic analysis of 142 MibHLHs: (**A**) intraspecific collinearity analysis of *Macadamia*, (**B**) statistics of bHLH duplication types in *Macadamia*, (**C**) collinearity analysis between *Macadamia* and *Arabidopsis thaliana*.

The circular structure is used to display the relationships between different bHLH genes. The yellow lines represent regions of sequence similarity or synteny among these genes. This visualization facilitates the identification of the genomic regions within *Macadamia* that have conserved gene order or sequence similarity, which is important for understanding the evolutionary history and genomic organization of the bHLH gene family in *Macadamia* (Figure 5A). Figure 5B is a pie chart that shows the statistics of different duplication types of the bHLH gene family in *Macadamia*. The different colors in the pie chart represent different duplication types. Dispersed (blue color) accounts for 66.9% of the duplication events. This type of duplication likely involves genes that are not close to each other in the genome after duplication. Proximal (green color) makes up 5.6% of the cases. Proximal duplication refers to the duplication of genes that are relatively close to each other in the genome. Singleton (purple color) represents 2.1% of the duplication types. Singleton duplication may involve genes that do not have a clear duplicate or have a very unique duplication pattern. Tandem (brown color) accounts for 8.5% of the cases. Tandem duplication means that duplicated genes are arranged one after another in the genome. Whole-genome duplication (WGD) or segmental duplication (cyan color) constitutes 16.9% of the duplication events. WGD or segmental duplication involves the duplication of a large segment of the genome, which can lead to the duplication of multiple genes at once. Figure 5C presents a collinearity analysis between *Macadamia* and *Arabidopsis thaliana*. The green bars at the bottom represent the chromosomes of *Macadamia*, labeled as Chr1, Chr2, Chr3, Chr4, Chr5, and chloroplast (ChrC) or mitochondrial chromosome (ChrM). The red lines and gray lines in the background represent the collinear regions between the *Macadamia* genome and the *Arabidopsis thaliana* genome. The red lines likely highlight the more significant or well-supported collinear relationships. This analysis is useful for understanding the evolutionary relationship between *Macadamia* and *Arabidopsis thaliana* at the genomic level, as it can reveal how genes or genomic regions have been conserved or rearranged during the evolution of these two species (Table S5).

**Figure 6:**
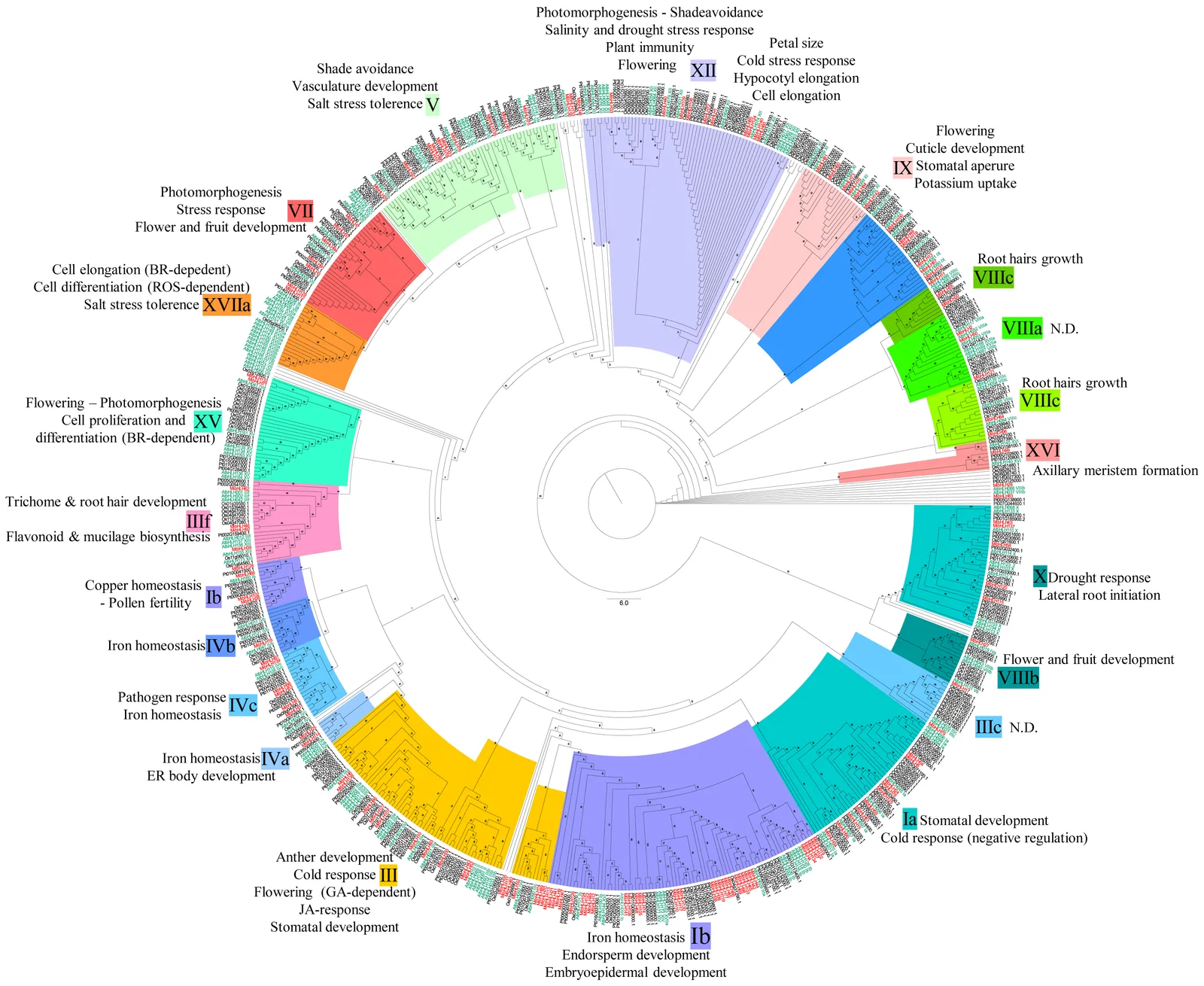
The subgroups of the MibHLH family and their potential functions. N.D., not determined.

The circular phylogenetic tree represents the 142 MibHLH transcription factor dividing into 21 subfamilies in *Macadamia* (Figure 6). Each subfamily is associated with specific biological functions and evolutionary relationships, as depicted by the branching patterns within each colored region. Each subfamily is linked to one or more biological processes, such as: Subfamily V (green) associated with shade avoidance, vasculature development, and salt stress tolerance; Subfamily VII (red) linked to photomorphogenesis, stress response, and flower and fruit development; Subfamily XV (cyan) related to flowering–photomorphogenesis as well as cell proliferation and differentiation (BR-dependent); Subfamily III (yellow) involved in another development, cold response, flowering (GA-dependent), JA-response, and stomatal development; Subfamily Ib (blue) associated with iron homeostasis, endosperm development, and embryopidermal development; and Subfamily VIIIc (green) linked to root hair growth. At present, the functions of the VIIIa and IIIc subfamilies have not been clearly defined (Table S6).

### 3.4. Relationship Between IIIf Subfamily Member MibHLH46 and the Flavonoid Pathway 

**Figure 7:**
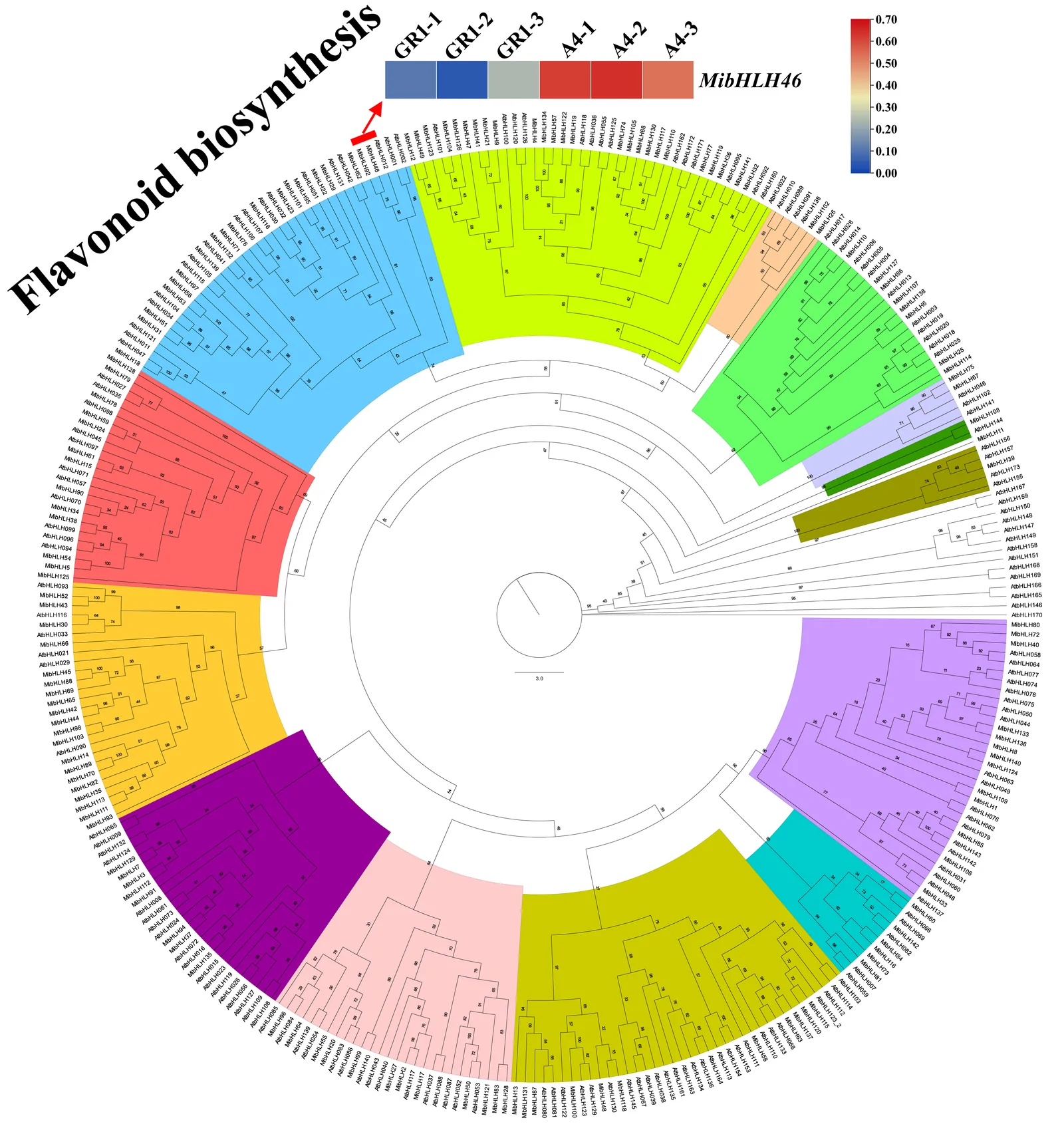
The *MibHLH46* gene showed significantly different expressions in ‘GR1’ and ‘A4’.

Expression analysis showed that *MibHLH46* may have a variable level of expression or activity in different samples. The red color (higher value, e.g., around 0.70) for samples in ‘A4’ and blue color (lower value, e.g., around 0.00) for samples in ‘GR1’ implies that *MibHLH46*’s expression is differentially regulated in these varieties. IIIf subfamily’s functions are related to trichome and root hair development, as well as flavonoid and mucilage biosynthesis. MibHLH46 TF is likely to be involved in the regulation of the flavonoid biosynthesis pathway (Figure 7, Table S7). 

**Figure 8:**
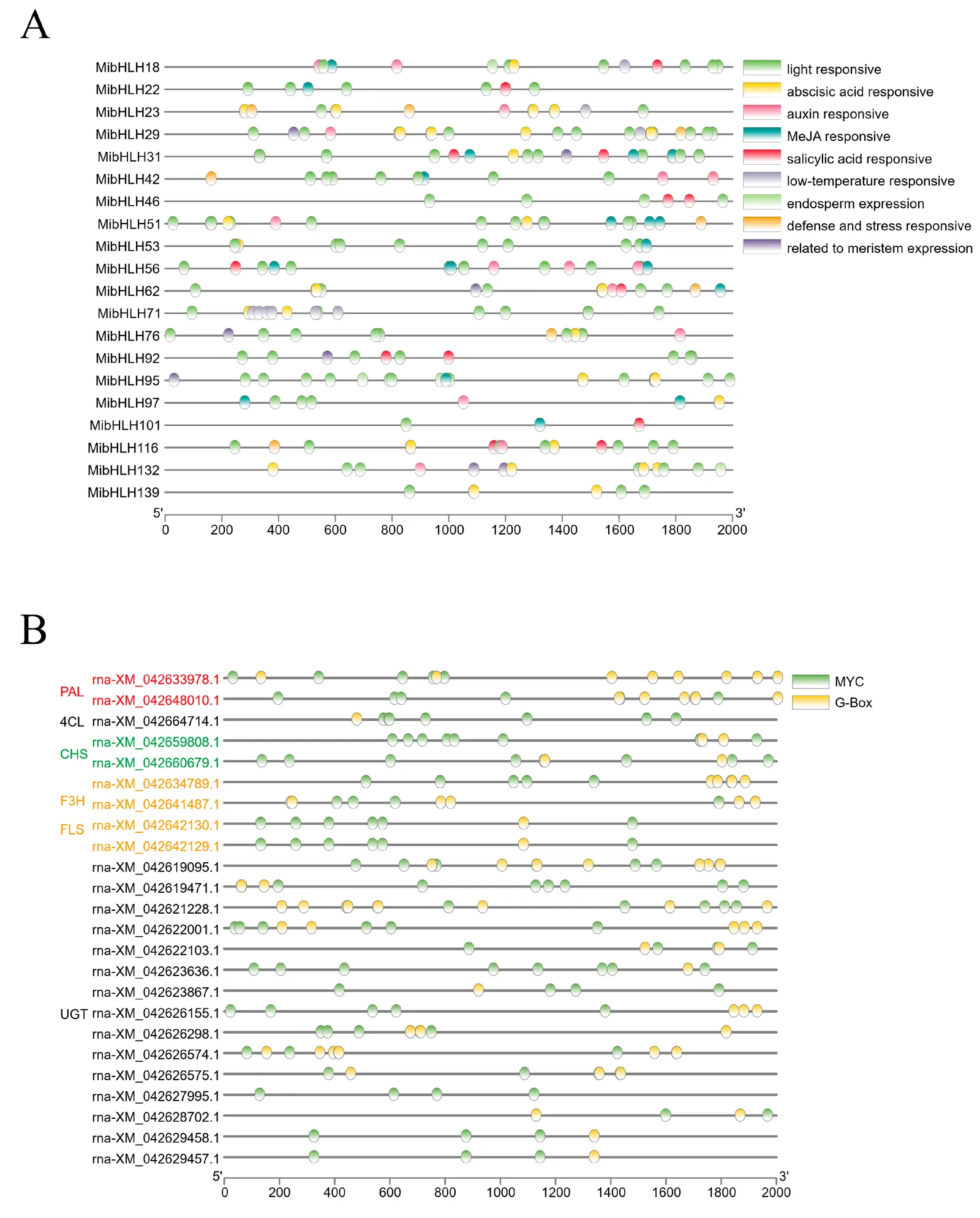
Promoter analysis of IIIf subgroup MibHLH family (**A**) and the key genes of fatty acid and flavonoid biosynthesis (**B**).

The promoter region of the *Macadamia MibHLH* IIIf subfamily gene (Figure 8A) contains diverse cis-regulatory elements that respond to various internal and external signals, such as light-responsive elements, abscisic acid (ABA)-responsive elements, auxin-responsive elements, methyl jasmonate (MeJA)-responsive elements, salicylic acid (SA)-responsive elements, low-temperature-responsive elements, endosperm expression elements, meristem expression-related elements, etc. The promoters of the *MibHLH46* gene only have light-responsive elements and SA-responsive elements, suggesting that MibHLH46 is mainly induced by light and SA signal. 

In Figure 8B, the promoters of the *Macadamia* genes involved in flavonoid and fatty acid synthesis contain two main bHLH-binding elements, namely, MYC and G-Box. MYC elements are recognized by bHLH transcription factors, especially those in the MYC subfamily, whilst G-Box elements are well-known cis-regulatory elements that bind to bHLH proteins to regulate gene expression. MibHLH proteins bind to MYC/G-Box elements in the promoters of flavonoid-related genes, including phenylalanine ammonia lyase (PAL), 4-coumarate: CoA ligase (4CL), chalcone synthase (CHS), flavonoid 3-hydroxylase (F3H), flavonol synthase (FLS), and UDP-glycosyltransferase (UGT), to activate their transcription. In summary, the bHLH gene promoter in *Macadamia* is a complex regulatory region that integrates multiple signals to control gene expression, while the bHLH-binding elements (MYC, G-Box) in flavonoid and fatty acid synthesis gene promoters play crucial roles in regulating these metabolic pathways (Table S8).

**Figure 9:**
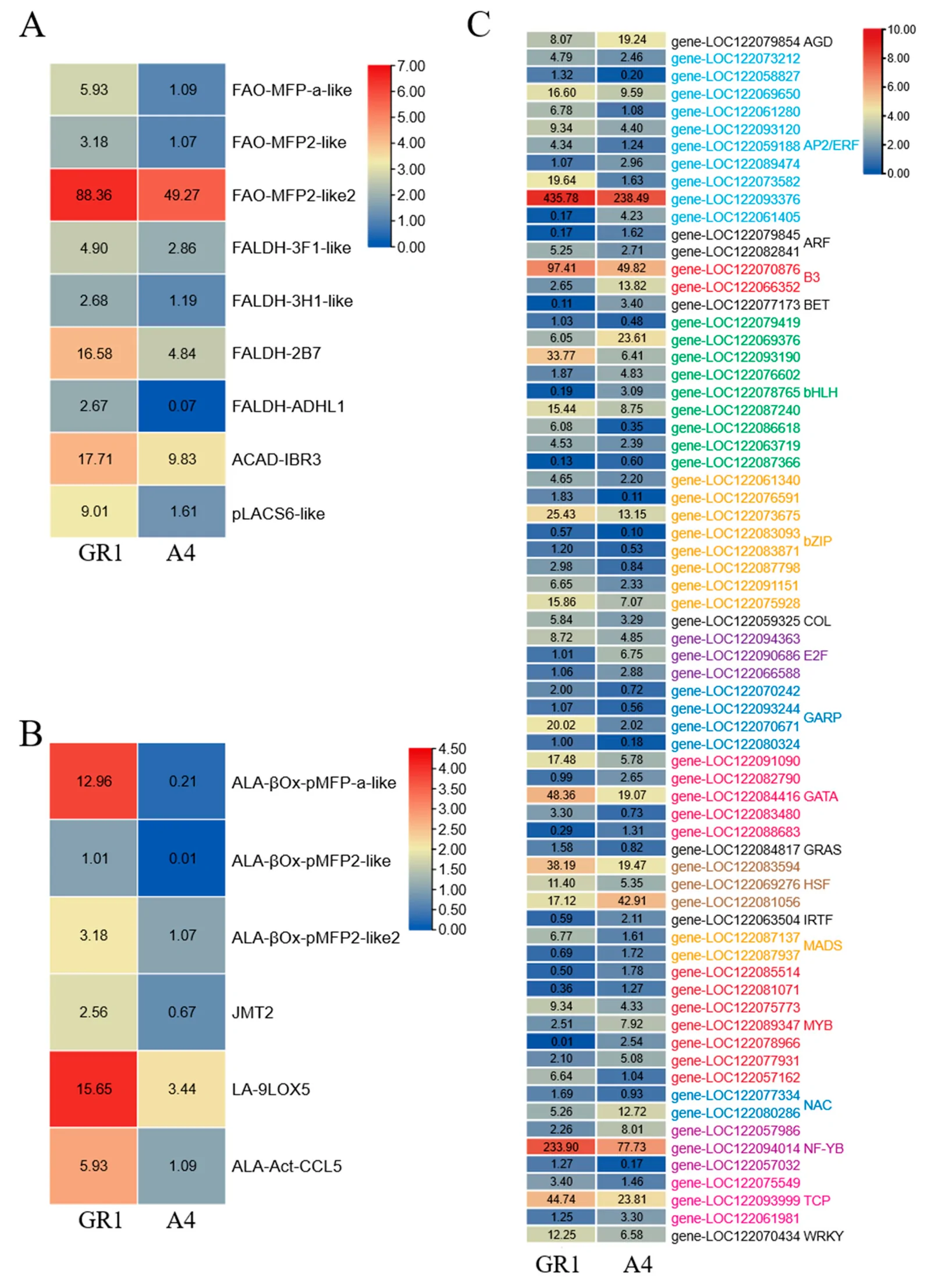
Differentially expressed genes between ‘GR1’ and ‘A4’: (**A**) 8 genes from fatty acid metabolism; (**B**) 6 genes from linoleic acid metabolism; (**C**) 69 transcription factor genes.

We analyzed the representative gene expression level of fatty acid metabolism, linoleic acid metabolism, and transcription factors in ‘GR1’ and ‘A4’. As shown in Figure 9A, the expression *ALA-βOx-pMFP-a-like* and *ALA-βOx-pMFP2-like* genes in ‘GR1’ were significantly higher than those in ‘A4’. For linoleic acid metabolism, similar to fatty acid metabolism, the *FAO-MFP-a-like* gene expression values were 5.93 in ‘GR1’ and 1.09 in ‘A4’. The *FAO-MFP2-like2* has higher values (88.36) in ‘GR1’ than in ‘A4’ (49.27) (Figure 9B). For transcription factors gene expression analysis, except for ARF-GAP DOMAIN (AGD), gene expressions in ‘GR1’ were lower than those in ‘A4’. Most transcription factor gene expressions were higher in ‘GR1’ than in ‘A4’, especially for bHLH TFs (Figure 9C, Table S9).

### 3.5. qRT-PCR Validation of Fatty Acid and Flavonoid Metabolism Genes

**Figure 10:**
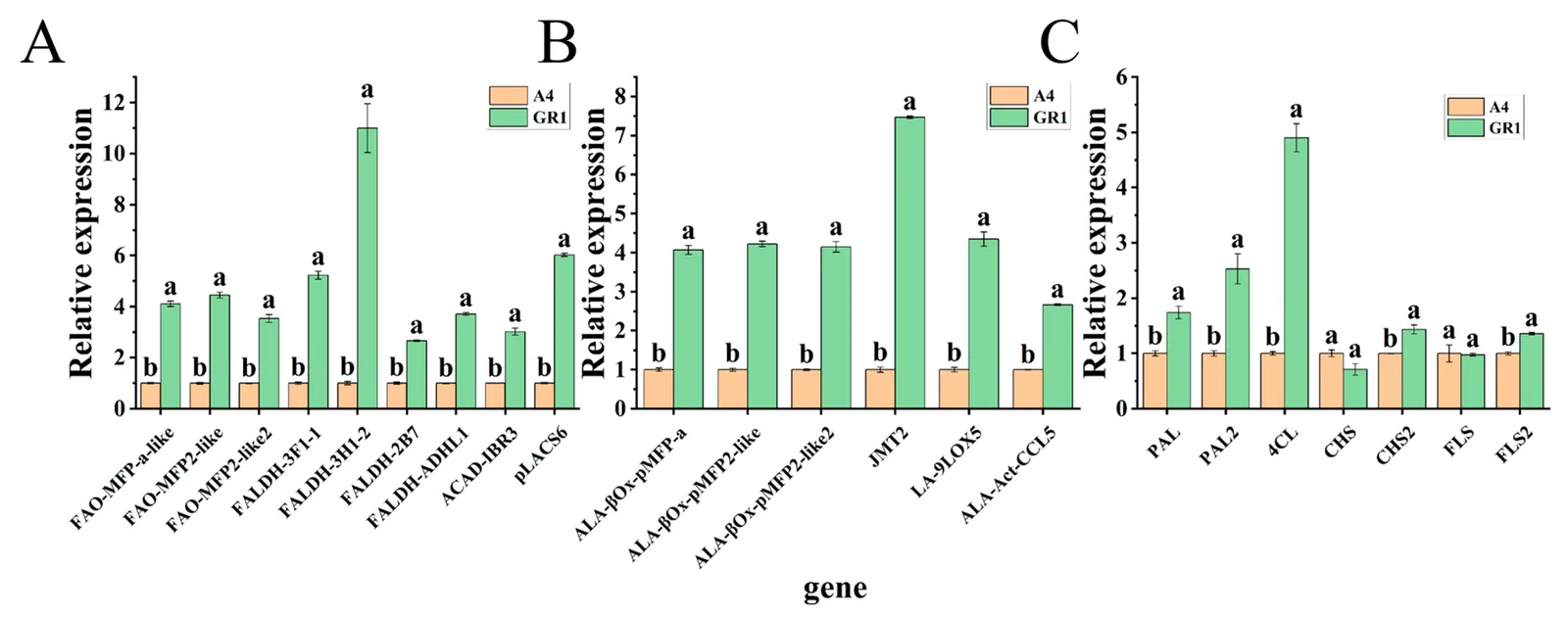
Validation of candidate gene expression in ‘GR1’ and ‘A4’ kernels by quantitative real-time PCR (qRT-PCR): (**A**) fatty acid oxidation and lipid metabolism genes; (**B**) α-linolenic acid metabolism-related genes; (**C**) flavonoid biosynthetic pathway genes. Relative expression was normalized to MiMADH and calculated via the 2^−ΔΔCT^ method with ‘A4’ functioning as the calibrator (mean ± SE, *n* = 3). Different lowercase letters indicate significant differences (*p* < 0.05).

To verify the transcriptomic data, the expression levels of key genes involved in fatty acid oxidation, α-linolenic acid metabolism, and flavonoid biosynthesis were examined via the qRT-PCR technique. Fatty acid oxidation and lipid metabolism genes were consistently and significantly up-regulated in ‘GR1’ kernels. The fatty acid oxidation multifunctional protein genes (*FAO-MFP-a-like*, *FAO-MFP2-like*, *FAO-MFP2-like2*) and the α-linolenic acid β-oxidation multifunctional protein gene (*ALA-βOx-pMFP-a*) showed 3.5- to 4.5-fold higher expression in ‘GR1’ than in ‘A4’ (*p* < 0.05). Within the fatty aldehyde dehydrogenase family, *FALDH-3H1-2* exhibited the strongest up-regulation, reaching an expression of about 11.0-fold higher than that of ‘A4’ (*p* < 0.05), while *FALDH-3F1-1* and *FALDH-ADHL1* were elevated by about 5.2-fold and 3.7-fold, and *FALDH-2B7* by about 2.7-fold. The *acyl-CoA dehydrogenase ACAD-IBR3* was about 3.0-fold higher, and the peroxisomal long-chain acyl-CoA synthetase *pLACS6* was about 6.0-fold higher (*p* < 0.05) (Figure 10A). The α-linolenic acid β-oxidation multifunctional protein paralogs (*ALA-βOx-pMFP2-like*, *ALA-βOx-pMFP2-like2*) were about 4.2-fold higher, *jasmonate O-methyltransferase JMT2* reached about 7.5-fold, *linoleate 9-lipoxygenase LA-9LOX5* reached about 4.4-fold, and *ALA-Act-CCL5* reached about 2.7-fold. Collectively, these data indicate that the transcriptional activity of the fatty acid β-oxidation and jasmonate biosynthesis pathways is substantially higher in GR1 kernels (Figure 10B).

Flavonoid biosynthetic genes displayed a divided pattern. Upstream genes *PAL*, its paralog *PAL2*, and *4CL* were significantly elevated in ‘GR1’ by about 1.7-fold, 2.5-fold, and 4.9-fold compared to those of ‘A4’, respectively (*p* < 0.05). However, the two pivotal enzymes, chalcone synthase (*CHS*) and flavonol synthase (*FLS*), showed no significant difference. This indicates that the flavonoid route is not coordinately up-regulated at every node; the increased transcription at upstream entry points and certain downstream branches is not reflected as a significant quantitative change at the CHS/FLS step (Figure 10C, Supplementary Table S10). In summary, the qRT-PCR results are highly concordant with the transcriptomic data. 

### 3.6. Molecular Pathway of bHLH-Mediated Fatty Acid Biosynthesis 

**Figure 11:**
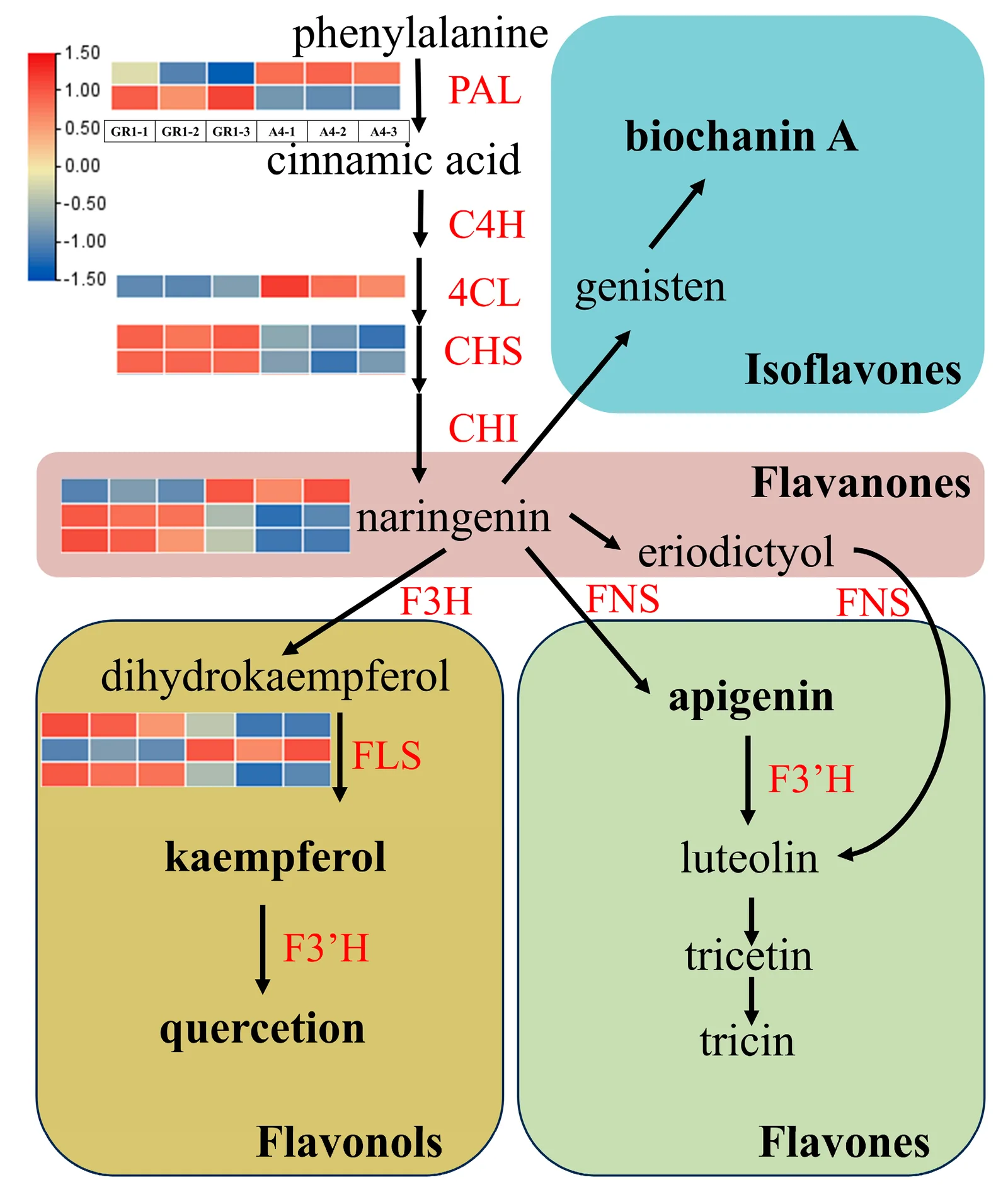
The DEGs of fatty acid and flavonoid biosynthesis pathways in ‘GR1’ and ‘A4’.

The metabolic pathway for flavonoid biosynthesis, starting from phenylalanine, implies cross-talk with fatty acid metabolism. The heatmap shows that gene expression differs between ‘GR1’ and ‘A4’. The main differences were found in the *PAL*, *4CL*, *CHS*, *F3H*, and *FLS* genes (Figure 11, Table S10). This indicates different fatty acid contents across *Macadamia* varieties, as flavonoid-mediated lipid metabolism varies.

## 4. Discussion

The high-quality oil content of *Macadamia* (*Macadamia* spp.) kernels, characterized by its rich composition of monounsaturated fatty acids (MUFAs), particularly oleic acid (C18:1) and the distinctive palmitoleic acid (C16:1, n-7), underpins its significant nutritional and economic value [2,36]. The biosynthesis and accumulation of these fatty acids (FAs) in developing kernels are tightly regulated at the transcriptional level [37]. Among the various regulatory players, the bHLH family of transcription factors (TFs) has emerged as a crucial component in modulating metabolic pathways in plants [19,38]. The bHLH protein family is one of the largest TF families in eukaryotes, defined by a conserved bHLH domain responsible for DNA binding and dimerization. In plants, bHLH TFs participate in diverse processes, including development [39], stress responses [40], secondary metabolism [41], etc. Critically, they are integral regulators of specialized metabolic pathways, often forming complexes with MYB and WD40 proteins (the MBW complex) to control flavonoid and anthocyanin biosynthesis [42,43]. Evidence suggests that their regulatory scope extends to primary metabolism, including lipid biosynthesis [20]. For instance, in *Arabidopsis*, bHLH factors like *ILR3/bHLH105* and *bHLH115* participate in iron homeostasis, which can indirectly influence FA desaturation [44]. Similarly, in *Nannochloropsis salina* (microalgae), overexpression of the bHLH TF *NsbHLH2* enhanced growth and fatty acid methyl ester (FAME) productivity [20]. These precedents establish a framework for investigating bHLH functions in *Macadamia* lipid synthesis.

Recent advancements in *Macadamia* genomics provide the foundation for identifying and characterizing bHLH TFs. High-quality chromosome-scale genomes have been assembled for species like *Macadamia integrifolia*, e.g., cultivar ‘GR1’ (about 792 Mb) [45], HAES 741 (about 745 Mb) [46], and *M. tetraphylla* about (750.87 Mb) [7]. These resources reveal gene family expansions related to defense and lipid metabolism. In *Arabidopsis*, *AtbHLH48* and *AtbHLH60* were related to flowering and hypocotyl elongation [47,48]. The mechanism by which bHLH TFs influence *Macadamia* kernel FA synthesis likely involves both the direct transcriptional regulation of pathway genes and participation in the broader hormonal or environmental signaling networks that modulate the lipid biosynthetic flux. 

The transcriptional differences between the two *Macadamia* varieties ‘GR1’ and ‘A4’ with significant differences in fatty acid content is reflected in multiple respects. At the overall transcriptional profile level, the two varieties are clearly separated in the PCA, indicating significant differences in gene expression patterns. At the differential expression level, a large number of genes are significantly up- or down-regulated between the two varieties, which provides a molecular basis for the difference in fatty acid content. At the functional enrichment level, the GO analysis shows that the differentially expressed genes are enriched in various molecular functions, cellular components, and biological processes related to metabolism, including lipid metabolism. At the pathway level, the KEGG analysis shows that the differentially expressed genes are enriched in lipid-metabolism pathways such as “fatty acid degradation” and other related metabolic pathways, such as carbohydrate metabolism and energy metabolism, which directly explains the molecular mechanism of the difference in fatty acid content between the two varieties (Figure 1 and Figure 2).

Complementing the transcriptomic and metabolomic differences, comparative profiling of mineral elements and phytohormones revealed that ‘GR1’ accumulates markedly higher levels of several elements (e.g., Fe, Ca, Mg) and abscisic acid (ABA) than A4 (Figure 1C). These mineral and hormonal divergences likely reflect the distinct nutritional and developmental statuses of the two varieties during kernel filling. Given that Fe, Mg, and Ca serve as cofactors for enzymes engaged in lipid metabolism, and that ABA is a central regulator of seed maturation and stress responses, such variations may accompany—or partly underlie—the divergent fatty acid and flavonoid accumulation between ‘GR1’ and ‘A4’, consistent with the broader hormonal signaling networks proposed to modulate lipid biosynthetic flux. The ABA content among macadamia varieties shows enormous differences, both in absolute concentration and in temporal dynamics. These differences are tightly linked to variety-specific physiological traits, higher ABA during flower bud differentiation promotes earlier and more successful flowering. Lower ABA during early fruit development reduces fruit abscission and improves fruit set. These findings highlight that ABA content is a major factor differentiating macadamia cultivars in terms of their reproductive success and yield potential, and that the specific timing, tissue localization, and hormone balance all contribute to the physiological outcome.

bHLH TFs typically bind to E-box (CANNTG) or G-box (CACGTG) motifs in the promoters of target genes. Several key enzymes in the *Macadamia* FA and triacylglycerol (TAG) assembly pathways are prime candidates for such regulation. For instance, the stearoyl-ACP desaturase (SAD) is a key enzyme introducing the first double bond. In *Macadamia*, *MiSAD* was cloned from *M. integrifolia* and shows high expression in leaves and kernels, peaking around 100 days after flowering—a period coinciding with active oil accumulation. While no specific bHLH regulator for *MiSAD* is reported here, in other plants, bHLH factors can regulate desaturase genes. Acyl-ACP thioesterases (FATs) enzymes terminate FA synthesis in the plastid, releasing FAs for further modification. In *Macadamia*, *FatA* and *FatB* thioesterases have been cloned and characterized. Notably, FatA from *M. tetraphylla* shows high specificity for 16:1-ACP, contributing to palmitoleic acid accumulation. The promoter regions of these *Fat* genes may contain cis-elements recognized by bHLH TFs, potentially linking the termination step to broader transcriptional programs (Figure 7).

The 142 *Macadamia* transcription factor genes exhibit diverse gene structures, a hallmark of complex gene families. They contain characteristic conserved protein motifs that define their functional domains. The pattern of these motifs is often correlated with phylogenetic grouping. The phylogenetic analysis successfully classifies these genes into distinct clades. Genes within the same clade generally share more similar gene structures and motif patterns than genes from different subfamilies. bHLH factors bind to the promoters of genes in the flavonoid pathway, e.g., *PAL*, *C4H*, *4CL*, *CHS*, *CHI*, *F3H*, *FLS*, *F3’H*, *FNS*, to activate or repress their expression (Figure 8). The results in Figure 9 indicate that there are significant differences in the expression of fatty acid synthesis genes and transcription factors between the two varieties (Figure 9). 

To verify the transcriptomic expression patterns, the 22 candidate genes were further examined by independent qRT-PCR (Figure 10). With the macadamia malate dehydrogenase gene *MiMADH* as the reference, the qPCR-derived relative expression levels were directionally consistent with the RNA-Seq fold-change calls for the majority of the validated genes. Fatty acid oxidation, α-linolenic acid metabolism, and flavonoid biosynthesis genes were confirmed to be up-regulated in ‘GR1’, mirroring the transcriptomic trends. This concordance between the sequencing-based and qPCR-based results strengthens the reliability of the differential expression calls and, together with the motif and correlation evidence above, supports a negative regulatory role of MibHLH46 in kernel lipid and flavonoid metabolism.

Although the promoter of MibHLH46 was found to contain predominantly light-responsive and SA-responsive cis-elements, with no canonical ABRE identified, this does not preclude its involvement in ABA-regulated processes. First, the E-box motifs (CANNTG) annotated as light-responsive elements in this promoter also belong to the broader G-box family, which includes ABRE sequences (PyACGTGG/TC), and have been reported to function as ABA-responsive cis-elements in other species. Second, ABA can regulate gene expression indirectly through MYB/MYC binding sites, which are present in this promoter. Third, as a subgroup IIIf bHLH protein, MibHLH46 may function as part of the MBW complex, where the MYB component mediates ABA responsiveness. Fourth, ABA may regulate the activity of this bHLH protein at the post-translational level. Future studies, including ABA treatment assays, electrophoretic mobility shift assays (EMSAs), and chromatin immunoprecipitation (ChIP), will be required to determine whether ABA directly regulates the transcription of this gene or whether the observed ABA-related effects are mediated through indirect mechanisms”.

A notable limitation of the present study is its exclusive reliance on a single pairwise comparison between the two macadamia varieties, ‘GR1’ and ‘A4’. Although these two varieties were selected for their contrasting fatty acid and flavonoid phenotypes, the observed differences in transcriptomic, metabolomic, and hormonal profiles may, in part, reflect a broader genetic background divergence between the two varieties rather than being specifically attributable to the regulatory activity of MibHLH46. Without the inclusion of additional varieties or near-isogenic lines, it remains challenging to disentangle the direct effects of MibHLH46 on target metabolic pathways from the pleiotropic consequences of numerous other genetic and epigenetic variations that distinguish ‘GR1’ from ‘A4’. Future studies should incorporate multi-variety comparisons or employ functional validation approaches, such as gene overexpression or knockout in a controlled genetic background, to confirm that MibHLH46 is a specific and causal regulator of fatty acid and flavonoid biosynthesis in macadamia kernels.

Based on this, we propose that the *MibHLH46* of the IIIf subfamily is a candidate negative regulator of fatty acid content, modulating the expression of the fatty acid synthase gene in *Macadamia*. It should be noted that the current study is based on correlative transcriptomic and metabolomic evidence, and direct functional validation of MibHLH46, e.g., through gene overexpression or knockout experiments, has not yet been performed. Therefore, the regulatory role of MibHLH46 in fatty acid and flavonoid biosynthesis remains to be confirmed through targeted experimental approaches in future studies.

## 5. Conclusions

A total of 142 MibHLH TFs were identified in ‘GR1’ and ‘A4’ through transcriptomic and metabolomic analyses. The macadamia bHLH family was classified into 21 subfamilies. Among them, MibHLH46 showed significant differential expression, as confirmed by the strong correlation between its expression and that of fatty acid synthesis-related genes. These expression patterns were further validated via independent qRT-PCR, which confirmed the up-regulation of fatty acid oxidation, α-linolenic acid metabolism, and flavonoid biosynthesis genes in ‘GR1’. It is evident that MibHLH46 may participate in the synthesis of flavonoids and fatty acids, possibly through transcriptional regulation of key biosynthetic genes such as MiPAL, although functional validation is required.

## Data Availability

The original contributions presented in this study are included in the article/Supplementary Material. Further inquiries can be directed to the corresponding author.

## Supplementary Materials

The following supporting information can be downloaded at https://www.mdpi.com/article/10.3390/biology15181589/s1: Table S1: RT-qPCR primers information; Table S2: Transcriptome sequencing reveals differentially expressed genes; Table S3: Analysis results of differentially expressed metabolites in the non-targeted metabolomics of the GR1 group and the A4 group; Table S4: MibHLH sequence information and protein physicochemical properties as well as localization; Table S5: MibHLH genomic genotype; Table S6: bHLH Subfamily; Table S7: Evolutionary basis of bHLH selection related to flavonoids; Table S8: Analysis of flavonoid-related bHLH cis-acting elements; Table S9: Information on genes with differential expression in fatty acid metabolism; Table S10: The differentially expressed genes for fatty acid and flavonoid biosynthesis in GR1 and A4; Table S11: Sequencing data statistics table.
